# Fibrin glue injection method for complex fistula after laparoscopic distal pancreatectomy: a case report

**DOI:** 10.1186/s13256-022-03406-7

**Published:** 2022-07-08

**Authors:** Hideki Izumi, Hisamichi Yoshii, Rin Abe, Masaya Mukai, Eiji Nomura, Hiroyasu Makuuchi

**Affiliations:** Department of Gastrointestinal Surgery, Tokai University Hachioji Hospital, 192-0032 Tokyo Hachioji, 1838 Ishikawa, Japan

**Keywords:** Pancreatic fistula, Fibrin glue injection, Distal pancreatectomy

## Abstract

**Background:**

Pancreatic fistula is the most problematic complication in pancreatectomy. Although drainage can be used to relieve this complication, pancreatic surgeons often encounter refractory pancreatic fistula. Fibrin glue injection, with the use of a twofold diluted solution B and a double-lumen tube, was found effective in treating this complicated pancreatic fistula.

**Case presentation:**

We report the case of a 64-year-old Japanese man who underwent laparoscopic distal pancreatectomy for pancreatic tail cancer. After initial drainage of the pancreatic fistula diagnosed 4 days postoperatively, on day 134, refractory pancreatic fistula was observed using contrast-enhanced computed tomography. We used fibrin glue injection, with a twofold diluted solution containing thrombin and calcium chloride and a double-lumen tube, for treating the refractory fistula; the fluid drainage was almost stopped with no fever or abdominal pain. No recurrence of pancreatic cancer has been observed since the procedure.

**Conclusions:**

Fibrin glue injection was effective for complicated pancreatic fistula after distal pancreatectomy. Using a twofold diluted solution B containing thrombin and calcium chloride and a double-lumen tube makes possible the thorough injection of fibrin glue.

## Background

Distal pancreatectomy is a well-established surgical procedure for treating pancreatic body and tail cancer. Despite the advancements in surgical techniques and perioperative management in recent years, numerous complication cases are still encountered during distal pancreatectomy. In particular, pancreatic fistula still accounts for a high percentage or 10%–40% of cases, causing serious complications such as intraabdominal abscess and hemorrhage [1, 2]. Although pancreatic fistula can often be relieved by drainage in general, complicated fistula formation with the development of refractory pancreatic fistula is occasionally encountered in some cases. Fibrin glue injection has been performed for treating refractory fistula, although there are various other medical procedures [3–6].

Herein, we report our experience of positive outcomes with fibrin glue injection using a twofold diluted thrombin injection method.

## Case presentation

**Fig. 1:**
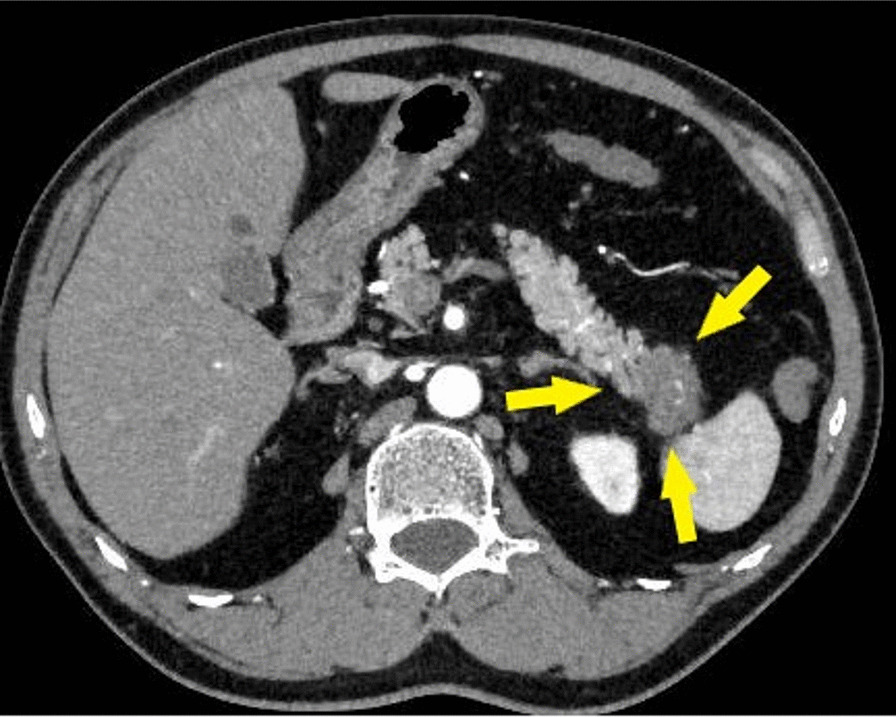
Preoperative contrast-enhanced computed tomography scan. The yellow arrow indicates a pancreatic cancer site

The patient was a 64-year-old Japanese man with a past medical history of high blood pressure. He was receiving conservative medical treatment because of the development of superior mesenteric artery dissection. A pancreatic tail tumor was detected in the periodical abdominal contrast-enhanced computed tomography (CT) (Fig. 1).

We performed laparoscopic distal pancreatectomy on the basis of the diagnosis of pancreatic tail cancer. In addition, a 5-min suppression and a 5-min resection were performed for the pancreatic cut end margin using a Powered Echelon 60 Green cartridge. Closed drainages were placed in the pancreatic stump and under the left diaphragm. The surgical time and hemorrhage volume were 267 min and 600 ml, respectively.

Histopathological findings showed invasive ductal carcinoma of the pancreas, Pt, 20 mm × 20 mm, and pStage IB (UICC, eighth edition).

On day 1 after surgery, the patient had a serum amylase level of 1303 U/L but high drain amylase levels (drainage of the pancreatic stump: 52,270 U/L, left subphrenic drainage: 6626 U/L). The color tone of the drainage was wine red.

On day 4 after surgery, his serum amylase level was 134 U/L, and he was diagnosed with pancreatic fistula on the basis of the drain amylase level (drainage of the pancreatic stump: 420 U/L, left subphrenic drainage: 865 U/L).

**Fig. 2:**
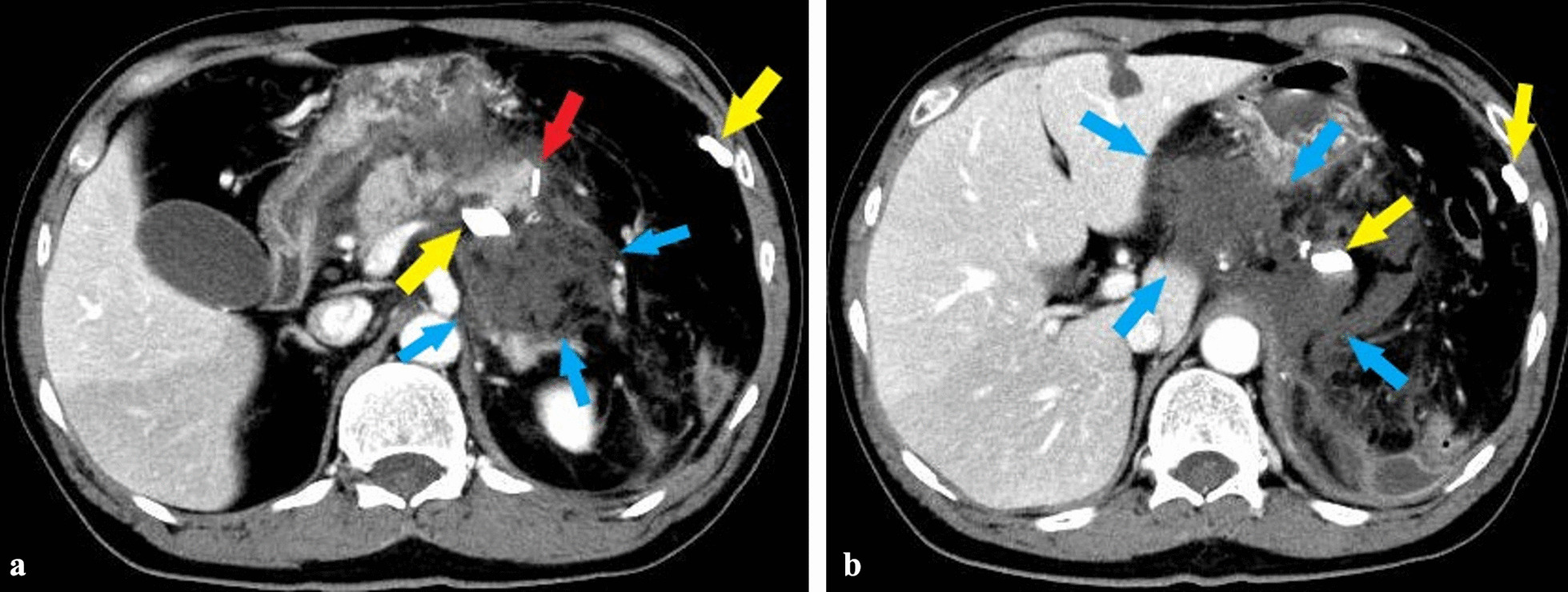
**a** Contrast-enhanced computed tomography examination on day 7 after surgery. The yellow, red, and blue arrows indicate the drain, stapler of pancreatic stump, and abscess formation of the pancreatic fistula, respectively. **b** Contrast-enhanced computed tomography examination on day 7 after surgery. The yellow and blue arrows indicate the drain and abscess formation of the pancreatic fistula, respectively

**Fig. 3:**
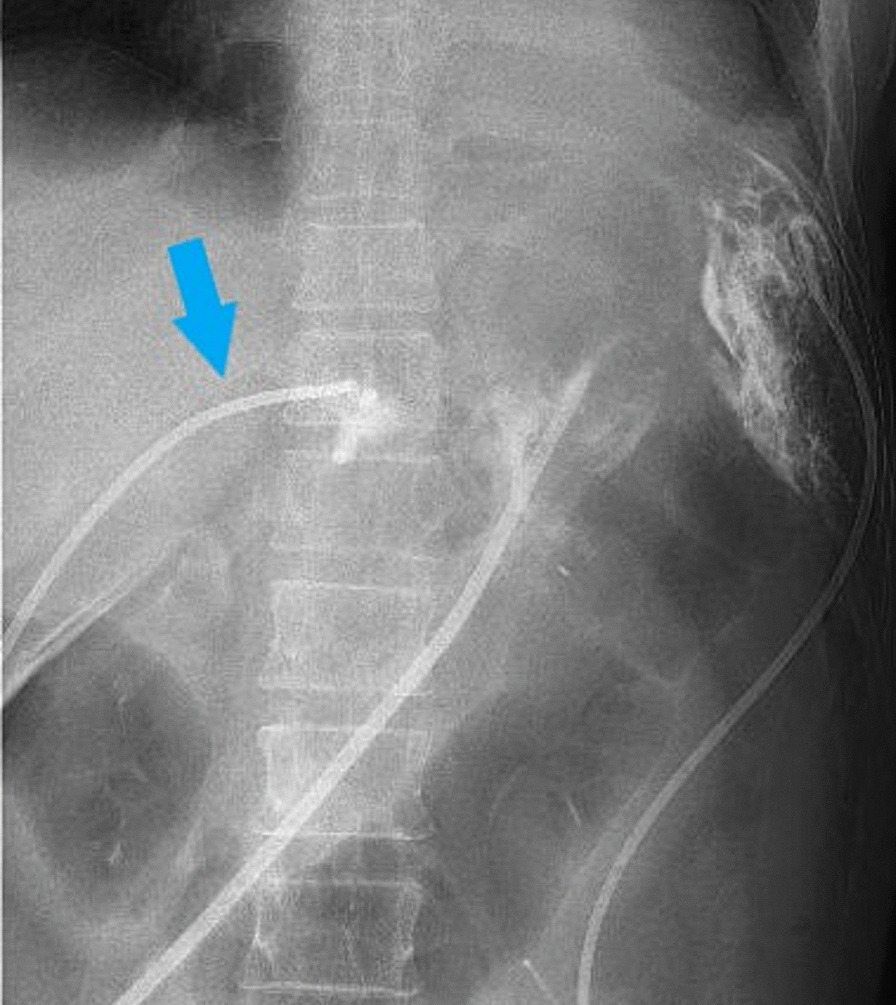
A percutaneous drainage tube was inserted on day 7 after surgery (blue arrow)

On day 7 after surgery, the patient developed a high inflammation level and persistent fever, due to which a contrast-enhanced CT scan was performed (Fig. 2a). The findings showed spread of abscess from the dorsal side of the stomach to the surrounding lateral segment of the liver (Fig. 2b), and hence, a drainage tube was inserted under echo guidance (Fig. 3).

On day 10 after surgery, the left subphrenic drain was removed as there was almost no drainage fluid.

On day 17 after surgery, the drainage tube inserted under echo guidance was removed. The patient’s condition was managed only by one pancreatic stump drain inserted during the surgery. Later, the drainage fluid volume was still approximately 50–100 ml.

**Fig. 4:**
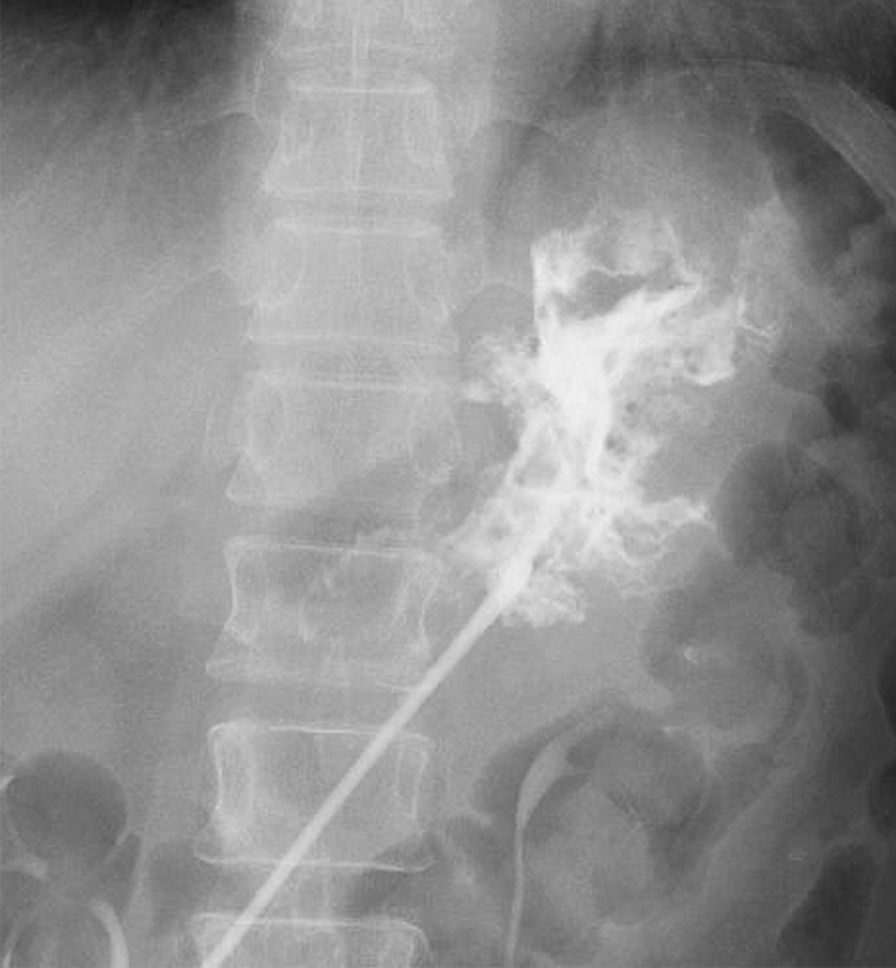
The abscess cavity was visualized by imaging from the drain on day 36 after surgery

On day 36 after surgery, a contrast agent administered from the drain revealed the image of abscess cavity (Fig. 4). However, we could not confirm a reduction in the abscess cavity. Nevertheless, we still made repeated attempts to adjust a drain location while periodically checking the size of the abscess cavity via imaging the drain.

No method other than drainage was used, and only drain changes were performed about once every 2 weeks.

**Fig. 5:**
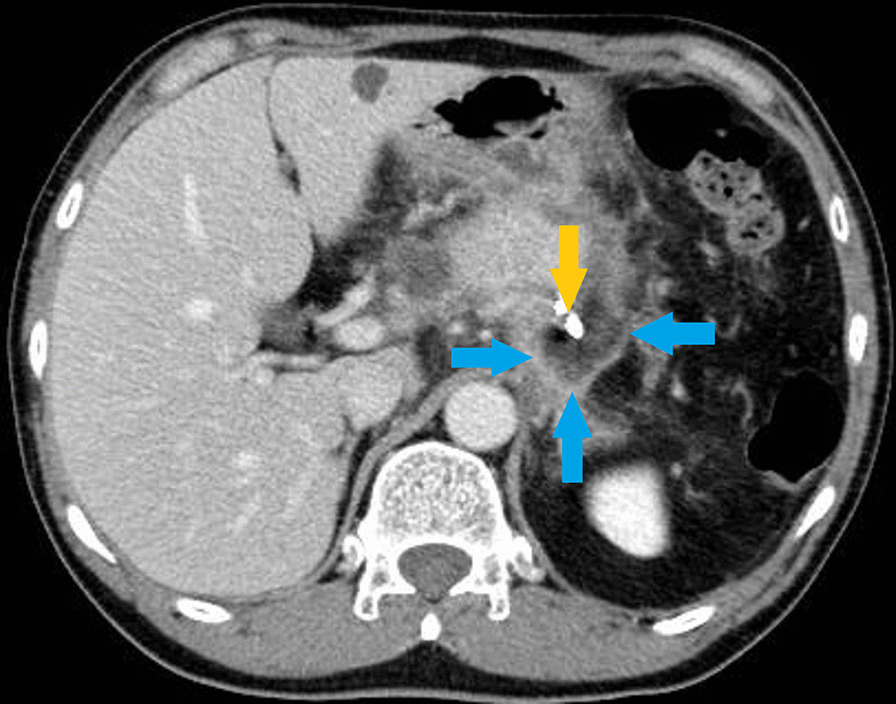
Contrast-enhanced computed tomography examination on day 134 after surgery. The yellow and blue arrows indicate the drain and the abscess cavity, respectively

**Fig. 6:**
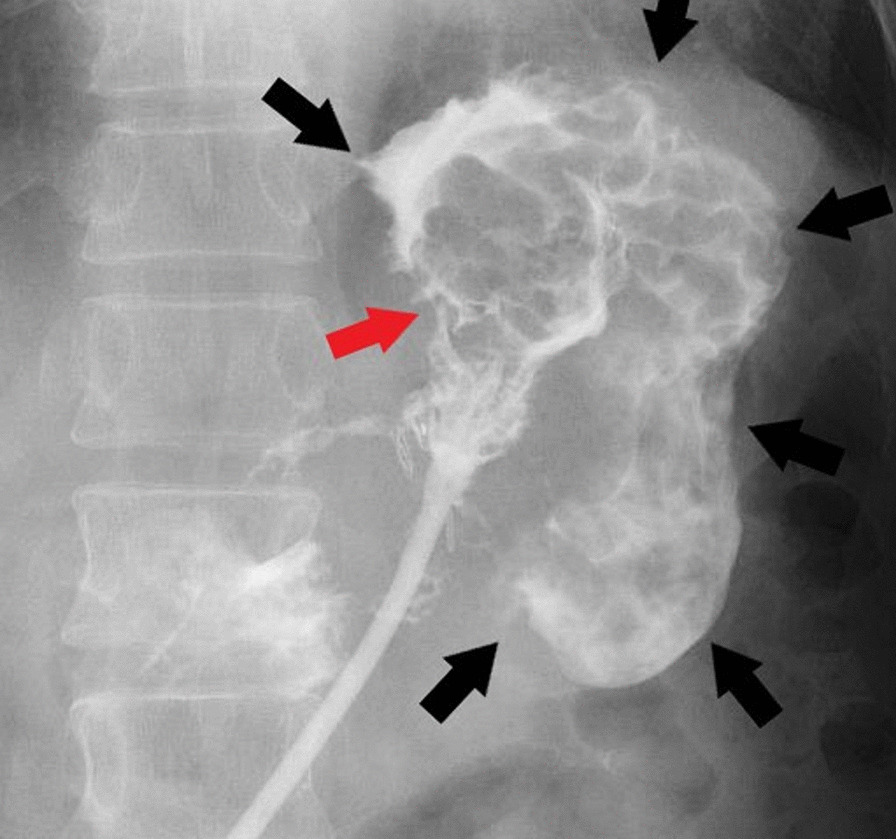
Drain imaging on day 134 after surgery. The contrast agent flows into the stomach from the red arrow portion. The black arrow indicates the line of the gastric wall

On day 134 after surgery, the patient developed fever, for which we conducted a contrast-enhanced CT that showed the abscess cavity around the drain (Fig. 5). After performing the contrast-enhanced CT imaging of the drain, we confirmed the presence of a communication to the stomach from the abscess cavity (Fig. 6).

On day 135 after surgery, fibrin glue (Beriplast P, ZLB Behring KK, Tokyo, Japan) was injected from the drain using Ring-McLean Sump Drainage Set-Straight (Cook Japan, Tokyo, Japan) as a double-lumen tube. We injected solution A containing fibrinogen from the primary route of the double-lumen tube and twofold diluted solution B containing thrombin and calcium chloride with normal saline solution from the other route. We injected both fluids at the same time while removing the drain over a period of about 5 seconds to ensure that the entire abscess cavity was covered with fibrin glue. Solution A and the twofold diluted solution B were simultaneously injected, after which the fluid drainage was almost stopped with no fever or abdominal pain at the time. The drain was removed 5 days after the injection as there was no increase in inflammation reaction even with the blood withdrawal.

Currently, 4 years has elapsed since the surgery, and the patient returns for a follow-up visit as an outpatient without any recurrence of pancreatic cancer.

## Discussion

Pancreatic fistula is the most problematic complication associated with pancreatectomy. According to recent reports, the frequency of the occurrence of postoperative pancreatic fistula in distal pancreatectomy is still high at 10%–40% [1, 2]. Pancreatic fistula may possibly become a trigger for serious complications such as intraabdominal abscess and hemorrhage. Drainage can be used as a basic method for dealing with pancreatic fistula. However, pancreatic surgeons often encounter refractory pancreatic fistula. Fibrin glue injection can be used for treating refractory pancreatic fistula with positive results.

Although there are several reports regarding the risk factors for pancreatic fistula, pancreatic thickness [7, 8] and hardness [9], BMI [10], and diabetes [11] can also be risk factors in distal pancreatectomy. There have been clinical trials for the treatment of pancreatic dissection and pancreatic resection/stump closure to prevent pancreatic fistula in distal pancreatectomy. The DISPACT trial, a large-scale multicenter study in Europe, was a randomized controlled trial (RCT) comparing pancreatic stump closure between an autosuture device and hand suturing, but there was no difference between those groups in terms of postoperative pancreatic fistula [12]. With the increasing application of laparoscopic pancreatectomy in recent years, pancreatotomy using a stapler has become more common [13]. Moreover, with the development of a cartridge equipped with polyglycolic acid mesh for reducing the incidence of pancreatic fistula, safety has also been confirmed in some studies reported till date [14, 15]. In addition, an RCT performed at the time of distal pancreatectomy to prevent pancreatic fistula by anastomosing the caudal pancreatic duct and the digestive tract of the remnant pancreas during pancreatectomy reported no significant difference between both sites [16]. Although the technique of preventive preoperative pancreatic duct stent placement has been described [17], it is not widely used because of a possibility of complications such as pancreatitis.

Several studies have demonstrated that fibrin glue injections are an effective treatment for refractory fistulas [3, 4]. It is believed that fibrin glue closes the fistula through two major phases of physiological coagulation [3]. In the first phase, it acts as a closing plug among tissues, resulting in rapid adhesion. The second phase involves promoting fibroblast cells into the fibrin glue, enhancing the growth of granulation tissue, and adsorbing various proteins such as fibronectin from the surrounding tissues. Thus, the glue is absorbed within 4 weeks and replaced by connective scar tissue [4, 5].

A problem associated with the fibrin glue injection method is that when fibrinogen solution is mixed with thrombin solution, the mixture would be coagulated in the catheter instantly, eventually causing catheter obstruction. In several cases, we have attempted to prevent coagulation in the catheter using a double-lumen catheter [5, 6]. Murakami* et al*. [18] reported that a complicated fistula could be filled in a condition where the coagulation time was extended without a change in tensile strength when the thrombin solution was diluted.

It is possible to achieve positive results by injecting a twofold diluted solution B containing thrombin and calcium chloride using a double-lumen tube. This method is considered as an effective treatment for refractory pancreatic fistula.

## Conclusions

Fibrin glue injection was effective for complicated pancreatic fistula after distal pancreatectomy. Using a twofold diluted solution B containing thrombin and calcium chloride and a double-lumen tube makes possible the thorough injection of fibrin glue.

## Data Availability

Not applicable.

## References

[1] Fujino Y. Perioperative management of distal pancreatectomy. World J Gastroenterol. 2015;21(11):3166–9. 10.3748/wjg.v21.i11.3166 PMC4363744 25805921

[2] Sell NM, Pucci MJ, Gabale S, Leiby BE, Rosato EL, Winter JM, Yeo CJ, Lavu H. The influence of transection site on the development of pancreatic fistula in patients undergoing distal pancreatectomy: a review of 294 consecutive cases. Surgery. 2015;157(6):1080–7. 10.1016/j.surg.2015.01.014 25791028

[3] Kurokawa T, Okushiba S, Kadoya M, Miyamoto D, Kurashima Y, Kitagami H, Ikeda J, Sunaga M, Shinzato Y, Ozawa T, *et al*. Selective occlusion with fibrin glue under fistuloscopy: seven cases of postoperative management for intractable complex fistulas. Endoscopy. 2002;34(3):220–2. 10.1055/s-2002-20294 11870573

[4] Eleftheriadis E, Kotzampassi, K. Therapeutic fistuloscopy: an alternative approach in the management of postoperative fistulas. Dig Surg 2002;19(3):230-235; discussion 236. 10.1159/000064218 12119527

[5] Ogunmola N, Wyllie R, McDowell K, Kay M, Mahajan L. Endoscopic closure of esophagobronchial fistula with fibrin glue. J Pediatr Gastroenterol Nutr. 2004;38(5):539–41. 10.1097/00005176-200405000-00015 15097445

[6] Brady AP, Malone DE, Tam P, McGrath FP. Closure of a duodenal fistula with fibrin sealant. J Vasc Interv Rad. 1993;4(4):525–7. 10.1016/s1051-0443(93)71910-7 8353350

[7] Sugimoto M, Gotohda N, Kato Y, Takahashi S, Kinoshita T, Shibasaki H, Nomura S, Konishi M, Kaneko H. Risk factor analysis and prevention of postoperative pancreatic fistula after distal pancreatectomy with stapler use. J Hepato-Bil Pancreat Sci. 2013;20(5):538–44. 10.1007/s00534-013-0596-0 23430057

[8] Okano K, Oshima M, Kakinoki K, Yamamoto N, Akamoto S, Yachida S, Hagiike M, Kamada H, Masaki T, Suzuki Y. Pancreatic thickness as a predictive factor for postoperative pancreatic fistula after distal pancreatectomy using an endopath stapler. Surg Today. 2013;43(2):141–7. 10.1007/s00595-012-0235-4 22782593

[9] Distler M, Kersting S, Rückert F, Kross P, Saeger HD, Weitz J, Grutzmann R. Chronic pancreatitis of the pancreatic remnant is an independent risk factor for pancreatic fistula after distal pancreatectomy. BMC Surg. 2014;14:54. 10.1186/1471-2482-14-54 PMC4184167 25127883

[10] Ferrone CR, Warshaw AL, Rattner DW, Berger D, Zheng H, Rawal B, Rodriguez R, Thayer SP, Fernandez-del Castillo C. Pancreatic fistula rates after 462 distal pancreatectomies: staplers do not decrease fistula rates. J Gastrointest Surg. 2008;12(10):1691–8. 10.1007/s11605-008-0636-2 PMC3806097 18704597

[11] Nakamura M, Shindo K, Ideno N, Ueda J, Takahata S, Nakashima H, Ohtsuka T, Shimizu S, Oda Y, Tanaka M. Prediction of pancreatic fistula by preoperatively assessable factors; retrospective review of unified operations by single surgeon. Hepatogastroenterology. 2014;61(131):834–7. 26176082

[12] Diener MK, Seiler CM, Rossion I, Kleeff J, Glanemann M, Butturini G, Tomazic A, Bruns CJ, Busch OR, Farkas S, *et al*. Efficacy of stapler versus hand-sewn closure after distal pancreatectomy (DISPACT): a randomised, controlled multicentre trial. Lancet. 2011;377(9776):1514–22. 10.1016/S0140-6736(11)60237-7 21529927

[13] Okano K, Kakinoki K, Suto H, Oshima M, Maeda N, Kashiwagi H, Yamamoto N, Akamoto S, Fujiwara M, Takama H, *et al*. Slow parenchymal flattening technique for distal pancreatectomy using an endopath stapler: simple and safe technical management. Hepatogastroenterology. 2010;57(102–103):1309–13. 21410078

[14] Hamilton NA, Porembka MR, Johnston FM, Gao F, Strasberg SM, Linehan DC, Hawkins WG. Mesh reinforcement of pancreatic transection decreases incidence of pancreatic occlusion failure for left pancreatectomy: a single-blinded, randomized controlled trial. Ann Surg. 2012;255(6):1037–42. 10.1097/SLA.0b013e31825659ef PMC3363360 22534422

[15] Park JS, Lee DH, Jang JY, Han Y, Yoon DS, Kim JK, Han HS, Yoon Y, Hwang D, Kang CM, *et al*. Use of TachoSil((R)) patches to prevent pancreatic leaks after distal pancreatectomy: a prospective, multicenter, randomized controlled study. J Hepato-Bil Pancreat Sci. 2016;23(2):110–7. 10.1002/jhbp.310 26681272

[16] Uemura K, Satoi S, Motoi F, Kwon M, Unno M, Murakami Y. Randomized clinical trial of duct-to-mucosa pancreaticogastrostomy versus handsewn closure after distal pancreatectomy. Br J Surg. 2017;104(5):536–43. 10.1002/bjs.10458 28112814

[17] Frozanpor F, Lundell L, Segersvärd R, Arnelo U. The effect of prophylactic transpapillary pancreatic stent insertion on clinically significant leak rate following distal pancreatectomy: results of a prospective controlled clinical trial. Ann Surg. 2012;255(6):1032–6. 10.1097/SLA.0b013e318251610f 22584629

[18] Murakami M, Tono T, Okada K, Yano H, Monden T. Fibrin glue injection method with diluted thrombin for refractory postoperative digestive fistula. Am J Surg. 2009;198(5):715–9. 10.1016/j.amjsurg.2008.10.026 19409528

